# Impact of Diversity in Training Resources on Self-Confidence in Diagnosing Skin Conditions Across a Range of Skin Tones: An International Survey

**DOI:** 10.3389/fped.2022.837552

**Published:** 2022-02-25

**Authors:** Danilo Buonsenso, Jo-Fen Liu, Dhurgshaarna Shanmugavadivel, Tessa Davis, Damian Roland

**Affiliations:** ^1^Department of Woman and Child Health and Public Health, Fondazione Policlinico Universitario A. Gemelli IRCCS, Rome, Italy; ^2^Dipartimento di Scienze Biotecnologiche di Base, Cliniche Intensivologiche e Perioperatorie, Università Cattolica del Sacro Cuore, Rome, Italy; ^3^Global Health Research Institute, Istituto di Igiene, Università Cattolica del Sacro Cuore, Rome, Italy; ^4^Academic Unit of Population and Lifespan Science, University of Nottingham, Nottingham, United Kingdom; ^5^Paediatric Emergency Department, Royal London Hospital, London, United Kingdom; ^6^Blizard Institute, Queen Mary University of London, London, United Kingdom; ^7^Paediatric Emergency Medicine Leicester Academic Group, Children's Emergency Department, Leicester Royal Infirmary, Leicester, United Kingdom; ^8^SAPPHIRE Group, Health Sciences, Leicester University, Leicester, United Kingdom

**Keywords:** child health, pediatrics, medical education, dermatology, ethics, diverity, skin tones

## Abstract

**Background:**

Medical images are invaluable in facilitating recognition of clinical signs. Recent studies highlight a lack of diversity of skin tone images used within medical education. However, there is a paucity of data on the impact of this on patient care.

**Aims:**

To investigate diversity in training resources used by users of an International online teaching platform and self-confidence in diagnosing skin conditions in all skin tones.

**Methods:**

Users of an online teaching platform (www.dftbskindeep.com) were invited to participate in a survey evaluating key points including geographical location, ethnicity, profession, specialty, years of experience, training resources and confidence in diagnosing skin conditions. Data analyses were performed using SPSS. Categorical variables were presented as proportions. Chi-squared or Fisher's exact tests were used to compare the distribution between groups as appropriate.

**Results:**

Of 600 participants, 74% reported training resources featuring predominantly white skin. Participants were “generally uncertain” in 43% cases, “sometimes uncertain but clinically safe” (52%), and “confident across a range of skin tones” in a minority (5%). Self-confidence was associated with location [higher in Africa (29%) and Latin America (11%), (*p* < 0.001)]; diversity of training resources [higher with a mix (10%) or darker tones (20%) (*p* < 0.001)]; clinical experience [6–10 (5%) or >10 years of practice (11%) (*p* < 0.001)] and specialty [highest in dermatologists (53%, *p* < 0.001)]. Self-confidence was lowest among pediatricians, emergency medicine and pediatric emergency medicine specialists (<5%).

**Conclusions:**

These data provide preliminary evidence that training resources used by healthcare professionals on a global scale may lack enough diversity on representation of skin images, and a lack of self-confidence in diagnosing pediatric skin conditions. Further work is needed to understand the impact on knowledge and patient care to ensure equitable healthcare for all.

## Introduction

Race, a recognized social determinant of health, has a profound impact on the health status of children, adolescents, emerging adults, and their families^1^,^2^. Although significant progress has been made in the last decade, there is still clear evidence of the negative impact of one's race on their health and wellbeing, through implicit and explicit biases, institutional structures, and interpersonal relationships (1). Unconsciously, the modern health system is contributing to feeding structural racism in healthcare. An example is demonstrated by skin color. Studies assessing diversity of skin representation among textbooks in the United States (US), Canada (2, 3) and in major scientific journals (4–7) have shown that the majority of images used within these educational materials are of light skin tones (defined in the oxford English dictionary as the color of the surface of someone's skin). Furthermore, descriptors of dermatological features also lack diversity; many descriptions of rashes focus on redness, pallor, purpura and cyanosis all of which are more difficult to recognize in darker skin tones or may not be present at all. Inequalities extend from education to research with evidence that children with darker skin tones have a lower probability of inclusion in clinical trials (8). The COVID-19 pandemic further highlighted these inequalities. Whilst evidence shows that Afro-Carribean and Asian patients experience a more severe illness course from COVID-19 (9), a systematic literature review demonstrated that articles describing the cutaneous manifestations of COVID-19 almost exclusively used clinical images from patients with light skin tones, with no single published images of darker skin tones (Fitzpatrick type V or VI) (10). This has the potential implication that patients, including children, with darker skin tones are more likely to experience a delayed or incorrect diagnosis compared to their lighter skin tone counterparts. Importantly, in a study assessing Afro-Caribbean patients' perception of their dermatology care, specialized knowledge in assessment and treatment of darker skin tones and hair was recognized by patients as a contributing factor for better care satisfaction (11).

The impact of the lack of representation in medical resources on the ability of healthcare professionals to recognize childhood skin conditions, however, has never been clearly documented. This is despite the American Academy of Pediatrics clearly advocating for rigorous research that examines, amongst others, “the impact of policy changes and community-level interventions on reducing the health effects of racism and other forms of discrimination on youth development” (1). This represents a large gap in the literature that needs to be urgently addressed.

This study aims to add to the dearth of data in this area by performing a pilot investigation of self-declared diversity of medical training resources used by healthcare professionals using an online teaching platform, and self-confidence in diagnosing skin conditions on different skin tones globally.

## Methods

An online platform was developed (www.dftbskindeep.com) hosting visual and teaching resources of dermatological conditions on a wide range of skin tones. The core team comprises international experts in the field of pediatric emergency medicine, pediatric infectious diseases, and pediatric dermatology, working in both Europe and Low-Middle Income Countries (LMICs).

The platform has been widely shared on social media and those who accessed the website were able to register and participate.

Participants were asked to log in and self-declare the following information (details on the information required and language are available on the registration page of the website; https://dftbskindeep.com/register/): continent of practice, ethnicity, profession, specialty, years of experience, training resources used within training (white skin, a mix of skin tones, darker skin tones only), and self-confidence in diagnosing skin conditions (generally uncertain if correct, sometimes uncertain but clinically safe, confident across range of skin tones). For this study purpose, responses collected between 26/08/2020 and 07/01/2021 were included. Since the aim of this pilot investigation is to assess the self-declared need of resources, and training materials, including a wider range of skin tones, we did not include a detailed classification of skin tones. Participants were made aware that non-identifiable demographic information would be shared as part of a research project prior to declaring the information. Given the methodology and the voluntary participation in the survey through a free website, ethical approvals were not deemed necessary. In the United Kingdom, where this study was conducted, the National Institute for Health Research (NIHR) deem health care professional research of this kind as not requiring formal ethical review (hra-decisiontools.org.uk).

### Statistical Analyses

Given the absence of previous data, a target sample size was set at 500 participants, in keeping with similar self-assessment and evaluative questionnaires run for Pediatric Acute Care topics of investigation (12, 13).

Data analyses were performed using IBM SPSS 26.0 for Windows (IBM Corp, Armonk, NY, USA). Categorical variables were presented as proportions, and Chi-squared or Fisher's exact tests were used to compare the distribution between groups as appropriate. A *p*-value of < 0.05 was deemed statistically significant in all analyses.

The Chi-Square Automatic Interaction Detector (CHAID) was used to further explore the interactions between respondent's characteristics and their confidence level. The dependent variable in the model was self-reported confidence (root node), and independent variables included participant's geographical location, ethnicity, experience, profession, specialty and majority training resources. At each step, the algorithm determines the best next split based on the magnitude of Bonferooni-adjusted χ^2^ statistics. Variable sub-categories are merged if they are not statistically different with respect to the outcome variable. The significance level for splitting nodes or merging categories was set at 0.05 (14). The variable that shows strongest relationships with the dependent variable appears in the first node of the decision tree. Node formation and segment configuration continue iteratively until no further splits can be performed, or the sample size in parent/child nodes is <30.

## Results

### Study Population

During the study period, 2,316 website visitors accessed the quiz registration page and 615 participants (26.5%) completed the survey. Fourteen were excluded for duplicate or incomplete data, one because the specialty reported was not eligible. Six hundred participants were included in the final analysis (Table 1). Participants responded from all continents, although Europe (56%) and Oceania (23%) were the most represented. The majority of participants were white/caucasian (69%). Four hundred and thirty-nine (73%) were clinicians, mostly pediatricians (37%), emergency doctors (22%) pediatric emergency doctors (12%) and a minority were dermatologists (3%). Just over half (56%) reported at least 6 years of practice since qualification.

**Table 1:** Characteristics of the survey respondents (*n* = 600).

	***n***	**Col%**
**Continent**		
Europe	337	56%
Oceania	140	23%
America	59	10%
Latin America	9	2%
Asia	41	7%
Africa	14	2%
**Ethnicity**		
White	411	69%
Asian/Oriental	124	21%
Black or African	29	5%
Hispanic or Latino	10	2%
Unclassified	26	4%
**Majority training resources**		
White skin	441	74%
A mix of skin tones	144	24%
Darker skin tones	15	3%
**Experience**		
Student	58	10%
1–2 years	65	11%
3–5 years	129	22%
6–10 years	188	31%
11 years +	159	27%
**Profession**		
Medic^*^	439	73%
Advanced nursing practitioner	47	8%
Nursing	60	10%
Paramedic and Other HCP^†^	21	4%
Other (not specified)	33	6%
**Specialty**		
Emergency medicine	129	22%
Emergency paediatrics	69	12%
Primary care	93	16%
Paediatrics	224	37%
Dermatology	17	3%
Other (not specified)	68	11%
**Confidence in diagnoses**		
Generally uncertain if correct	259	43%
Sometimes uncertain but clinically safe	309	52%
Confident across range of skin tones	32	5%

* *Including medical doctors/primary care practitioner (all grades) and medical student (n = 3), physician associate or assistant (4) and physician assistant-student (1)*.

† *Including paramedic (16) and clinical pharmacist (1), pharmacist (1), physiotherapist (1), podiatrist (1) and respiratory therapist (1)*.

Participants were asked to report if the majority of training resources used during their training were inclusive of materials with a range of skin tones. Four hundred and forty-one (74%) reported that only white skin was usually represented, while resources including a mix of skin tones (24%) or other skin tones (3%) were less represented. The diversity of training resources used across continents and according to ethnicity are shown in Supplementary Table 1. In Latin America and Africa, resources with only white skin images were reported in 44 and 29% respondents, respectively.

### Self-Confidence in Diagnoses of Skin Conditions Across Different Skin Tones

Overall, 43% of participants reported that they are “generally uncertain if correct”, 52% were “sometimes uncertain but clinically safe”, with only 5% “confident across a range of skin tones”.

Table 2 and Supplementary Figures 1, 2 show the correlations between demographics and reported self-confidence in diagnosing skin conditions across different skin tones. Self-confidence level was associated with geographical location, diversity of training resources used, specialty and years of experience. Those living in Africa (4/14, 29%) and Latin America (1/9, 11%) were most confident across a range of skin tones which was statistically significant (*p* < 0.001). Confidence was higher in those who trained using a mix of skin tones (15/144, 10%) or darker skin tones only (3/15, 20%) (*p* < 0.001). Confidence was also highest in those with more clinical experience [6–10 (9/188, 5%) or >10 years of practice (18/159, 11%) (*p* < 0.001)], and by specialty [highest in dermatologists (9/17, 53%, *p* < 0.001)]. Self-confidence was very low among pediatricians, emergency medicine and pediatric emergency medicine specialists (<5%). Ethnicity did not seem to affect self-confidence (p > 0.05).

**Table 2:** Self-reported confidence in diagnosis.

	**Confident across range of skin tones (*****n*** **=** **32)**		**Sometimes uncertain but clinically safe (*****n*** **=** **309)**		**Generally uncertain if correct (*****n*** **=** **259)**		***p*-value**
	***n***	**Row %**	***n***	**Row %**	***n***	**Row %**	
**Continent**							<0.001
Europe	20	6%	178	53%	139	41%	
Oceania	1	1%	58	41%	81	58%	
America	3	5%	36	61%	20	34%	
Latin America	1	11%	6	67%	2	22%	
Asia	3	7%	23	56%	15	37%	
Africa	4	29%	8	57%	2	14%	
**Ethnicity**							0.257
White	17	4%	206	50%	188	46%	
Asian/Oriental	9	7%	67	54%	48	39%	
Black or African	4	14%	15	52%	10	34%	
Hispanic or Latino	1	10%	6	60%	3	30%	
Unclassified	1	4%	15	58%	10	38%	
**Majority training resources**							<0.001
White skin	14	3%	214	49%	213	48%	
A mix of skin tones	15	10%	87	60%	42	29%	
Darker skin tones	3	20%	8	53%	4	27%	
**Experience**							<0.001
Student	1	2%	10	17%	47	81%	
1–2 years	0	0%	29	45%	36	55%	
3–5 years	4	3%	59	46%	66	51%	
6–10 years	9	5%	105	56%	74	39%	
11 years +	18	11%	106	67%	35	22%	
**Profession**							0.206
Medic^*^	28	6%	229	52%	182	41%	
Advanced nursing practitioner	0	0%	28	60%	19	40%	
Nursing	2	3%	28	47%	30	50%	
Paramedic and Other HCP^†^	1	5%	6	29%	14	67%	
Other	1	3%	18	55%	14	42%	
**Specialty**							<0.001
Emergency medicine	3	2%	54	42%	72	56%	
Emergency paediatrics	2	3%	46	67%	21	30%	
Primary care	3	3%	55	59%	35	38%	
Paediatrics	9	4%	122	54%	93	42%	
Dermatology	9	53%	8	47%	0	0%	
Other	6	9%	24	35%	38	56%	

* *Including medical doctors/primary care practitioner (all grades) and medical student (n = 3), physician associate or assistant (4) and physician assistant-student (1)*.

† *Including paramedic (16) and clinical pharmacist (1), pharmacist (1), physiotherapist (1), podiatrist (1) and respiratory therapist (1)*.

**Figure 1:**
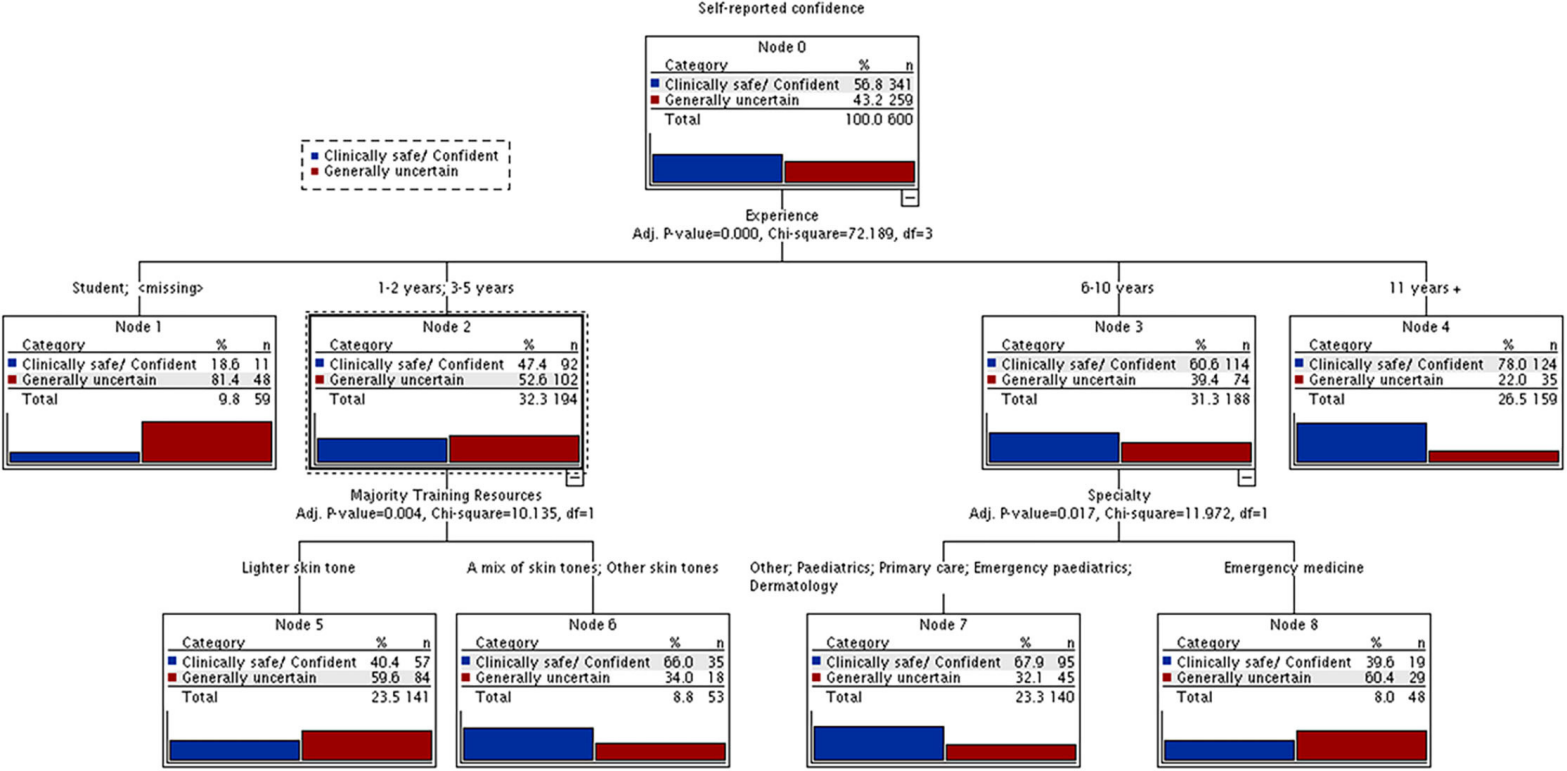
CHAID (Chi-square Automatic Interaction Detector) was used to explore relationships between self-reported confidence and other factors. Blue bar, sometimes uncertain but clinically safe + confident across range of skin tones; Redbar, generally uncertain if correct.

Figure 1 and Supplementary Table 2 explore the relationships between self-reported confidence in diagnosing skin conditions across different skin tones and other factors. Years of clinical experience was the main parameter positively affecting self-confidence, with those having at least 10 years of experience being the most self-confident (*p* < 0.0001). The impact of training resources was also analyzed in those groups whose self-confidence according to years of experience was borderline. Those with 1–5 years of experience were generally uncertain in 52.6% of cases. The type of resources used during training significantly affected self-confidence, with those who reported a mix of skin tones or exclusively darker skin tone resources being significantly more self-confident (*p* = 0.004). Furthermore, by specialty, those working in emergency medicine were least confident (*p* = 0.01).

## Discussion

In this study, we asked users of an international teaching online platform to self-declare the diversity in training resources used during their education and their self-confidence in diagnosing skin conditions in a range of skin tones. To our knowledge, this is the first international study to answer this crucial question in such a diverse cohort of healthcare professionals from different ethnic, cultural and professional backgrounds.

These data highlight that most participants declared they predominantly trained using educational materials featuring light skin tones. Although Latin America and Africa used more representative resources, there were still a large percentage of respondents from these areas who reported training resources representing only white skin. These data are, unfortunately, in line with other published data. Of 1,381 images assessed in the New England of Medicine (4), 80% of images depicted white skin and 18% depicted non-white skin (*p* < 0.0001), reporting also considerable heterogeneity in the percentage of non-white medical images published from different geographic regions and specialties (ranging from 0 to 67%). The same group published a study in 2019 (5) which assessed diversity in published skin images in the New England Journal of Medicine and others from 1992 and 2017. Of 24,209 color photographs and 1,671 color graphics, only 22% of photographs were of darker skin tones. Importantly, they did report an increase in the use of darker skin tone images over time (*r* = 0.086, *p* < 0.001) and an association of darker skin tone images with international authors (*r* = 0.12, *p* < 0.001). A similar underrepresentation of darker skin tone was reported in training textbooks in Canada and US, respectively (2, 3). Furthermore, in two studies assessing images in plastic surgery, only 20% of images represented darker skin tones (6, 7). Recently, another study showed that darker skin phenotypes are underrepresented in the UWorld Step 2 QBank, a popular study tool for medical students seeking US residencies (15).

An important finding of this study is the self-reported confidence and the factors affecting it. In general, only 5% of respondents felt confident across a range of skin tones with 43% being generally uncertain if correct. As expected, working experience significantly affected self-confidence (*p* < 0.001). However, a more interesting finding was represented by the link between type of training resources and self-confidence. Only 3% of those using training resources with only white-skin images felt confident in diagnosing skin conditions on different skin tones (*p* < 0.001). The use of more inclusive resources positively correlated with increased confidence especially when taking into account the participant's level of experience (Figure 1).

These data also highlighted that medical specialty affected reported self-confidence. Whilst the finding that dermatologists felt most confident was expected (none reported to be usually uncertain), both pediatricians and emergency physicians reported being generally uncertain (42 and 56%, respectively). Data analysis according to years of experience, in the group “6–10 years”, being an emergency physician was significantly associated with less self-confidence (*p* = 0.017). These findings need further validation but, if confirmed, will need a review of training programs and a development of more inclusive resources. This is particularly relevant since pediatricians and emergency physicians interact with a wide range of different ethnicities in emergency departments. However, although our findings are new for the pediatric field, dermatologists have already declared in previous studies that they did not feel their education had adequately prepared them to diagnose and manage skin disease in Black patients (16). Also, a recent study involving medical students showed that they were less accurate in diagnosing squamous cell carcinoma, atopic dermatitis, and urticaria in patients with skin of color (17).

Our study has some limitations. Firstly, although the platform is very-well established in the pediatric field with a wide international audience, the survey may have had a non-homogenous distribution, which could have affected participation from LMICs. In fact, participants from Latin America and Africa, and those with non-white ethnicity, were poorly represented. Secondly, health care providers with an interest in dermatology and diagnosis might have been more likely to participate and, therefore, could represent a selection bias. However, our survey still reports the largest data available on this topic globally and it seems improbable that a larger response would significantly reverse the trends. Thirdly, participants may have reported their self-confidence with a “pediatric-lens”, reporting their skills in recognizing only pediatric skin conditions. This may have affected the emergency physicians' self-confidence compared to their confidence in diagnosing adult skin conditions. We also acknowledge the known limitations of self-reporting of confidence and hope this study will represent the preliminary step for a future prospective study assessing ability in diagnosing skin conditions across different skin tones in an objective manner. In this regard, using English as the sole language can be a further limitation, however the questions were relatively simple. Lastly, another important limitation of this study is the self-assessment of diversity of medical resources, for a number of reasons: we could not directly address the skin representation in training resources used; also, the concept of diversity is subjective and our investigation relies on an implied subjective judgment about what counts as enough diversity. Nevertheless, despite these limitations, our findings are in line with recent positions from major societies, highlighting the urgent need of addressing equality and diversity in medical education. A recent viewpoint from US dermatologists called for a dermatologic curricula that incorporate concepts of antiracism, implicit bias, cultural humility, health disparities, and skin-of-color education into journal clubs, grand rounds presentations, resident lectures, and direct patient exposure (18–20) and similar devices have been raised by the American Academy of Pediatrics (21) and by European paediatricians (22).

In conclusion, our study provides preliminary data that training resources used by healthcare professionals on a global scale may lack enough diversity in representation of skin images. Importantly, our findings also provide preliminary evidence that this lack of diversity can possibly affect self-confidence in diagnosing skin conditions on a variety of skin tones, which has the potential to impact child health outcomes from correct and timely diagnosis of skin conditions on children and young people. These data need urgent prospective validation using more rigorous methodology, in order to provide evidence to redefine training programs of healthcare professionals worldwide.

## Data Availability Statement

The raw data supporting the conclusions of this article will be made available by the authors, upon request without undue reservation.

## Ethics Statement

Ethical review and approval was not required for the study on human participants in accordance with the local legislation and institutional requirements. Written informed consent from the participants' legal guardian/next of kin was not required to participate in this study in accordance with the national legislation and the institutional requirements.

## Don't Forget the Bubbles

The DFTB Skin Deep core team: Nikki Abela, Michelle Alisio, Safeena Afzal, Oke Obiuwevbi, Nensi Parekh, Alyah Seif, Keir Shiels, Aarani Somaskanthan, Kaylita Chantiluke, Michelle Arora, Holly Wakefield, and Rebecca Platt.

## Author Contributions

DB, DS, TD, and DR designed the study. J-FL and DS analyzed the data. DB drafted the manuscript which was reviewed and edited by DS, J-FL, TD, and DR. All authors contributed to the article and approved the submitted version.

## Funding

This work was supported by Surf4Children, a no profit organization is leading a health project in Sierra Leone and was involved in images collection and supported the open access fee, without affecting the study design.

## Conflict of Interest

TD and DR are executive committee members of Don't Forget the Bubbles. The remaining authors declare that the research was conducted in the absence of any commercial or financial relationships that could be construed as a potential conflict of interest.

## Publisher's Note

All claims expressed in this article are solely those of the authors and do not necessarily represent those of their affiliated organizations, or those of the publisher, the editors and the reviewers. Any product that may be evaluated in this article, or claim that may be made by its manufacturer, is not guaranteed or endorsed by the publisher.

## Footnotes

^1^ https://www.nejm.org/race-and-medicine

^2^ https://www.thelancet.com/racial-equality
