# Supplementary material for: Impact of Diversity in Training Resources on Self-Confidence in Diagnosing Skin Conditions Across a Range of Skin Tones: An International Survey
Source: Front Pediatr. 2022 Feb 25;10:837552. doi: 10.3389/fped.2022.837552 (PMC8916608; doi:10.3389/fped.2022.837552)
Supplement: Supplementary file 1 [file Data_Sheet_1.PDF]

Impact of diversity in training resources on self-confidence in diagnosing skin conditions across a range of skin tones: an international survey

Supplementary material

	White skin (n=441)		A mix of skin tones (n=144)		Darker skin tones (n=15)		p-value
**Continent**							
Europe	273	81%	59	18%	5	1%	<0.001
Oceania	106	76%	33	24%	1	1%	
America	42	71%	14	24%	3	5%	
Asia	12	29%	26	63%	3	7%	
Africa	4	29%	7	50%	3	21%	
Latin America	4	44%	5	56%	0	0%	
**Ethnicity**							
White	326	79%	79	19%	6	1%	<0.001
Asian/Oriental	75	60%	44	35%	5	4%	
Black or African	16	55%	9	31%	4	14%	
Hispanic or Latino	6	60%	4	40%	0	0%	
Unclassified	18	69%	8	31%	0	0%	

Supplementary table 1. Diversity of training resources according to continent of practice and ethnicity.

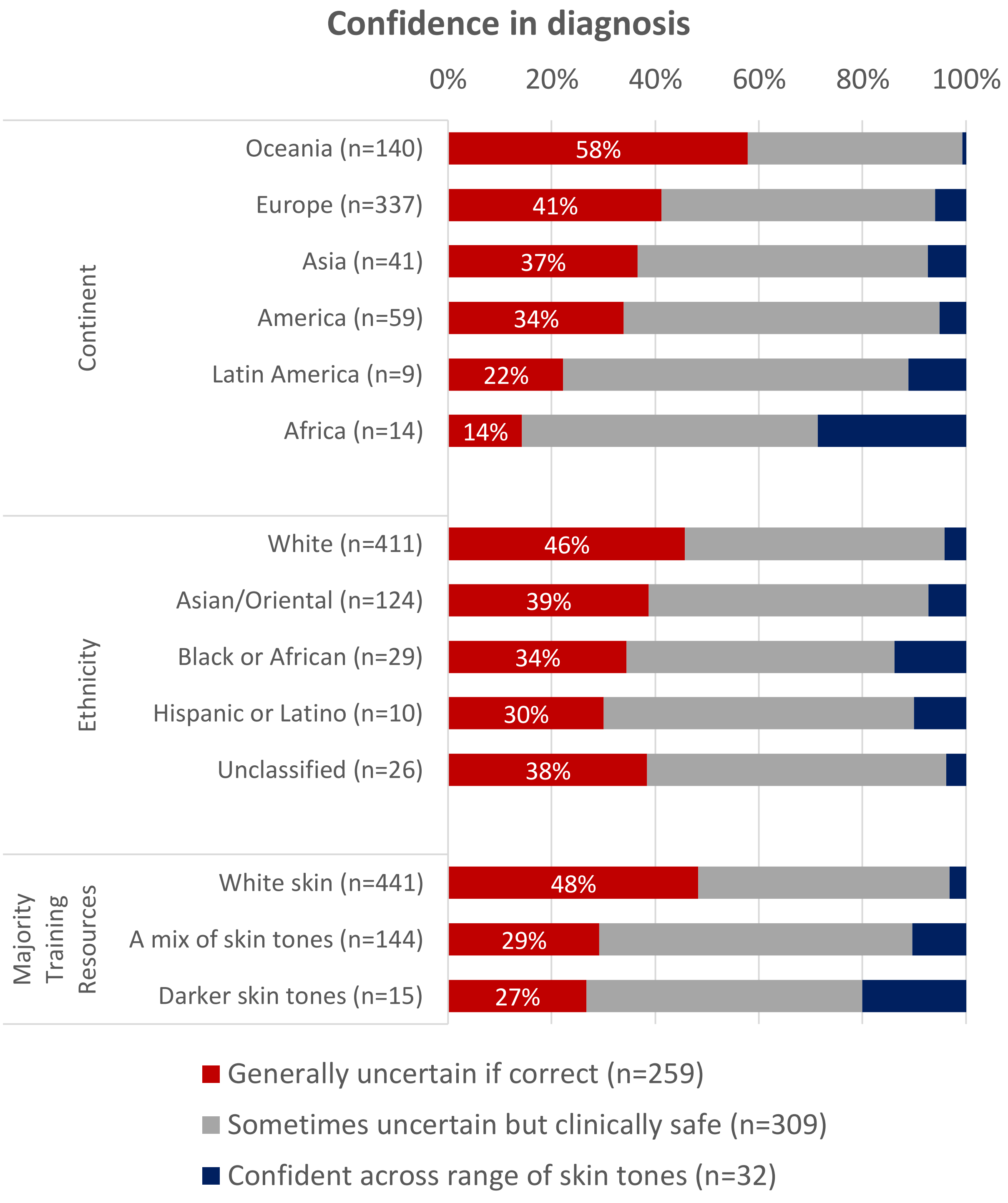

Supplementary figure 1. Self-confidence in diagnoses according to continent of practice, ethnicity and major training resources used.

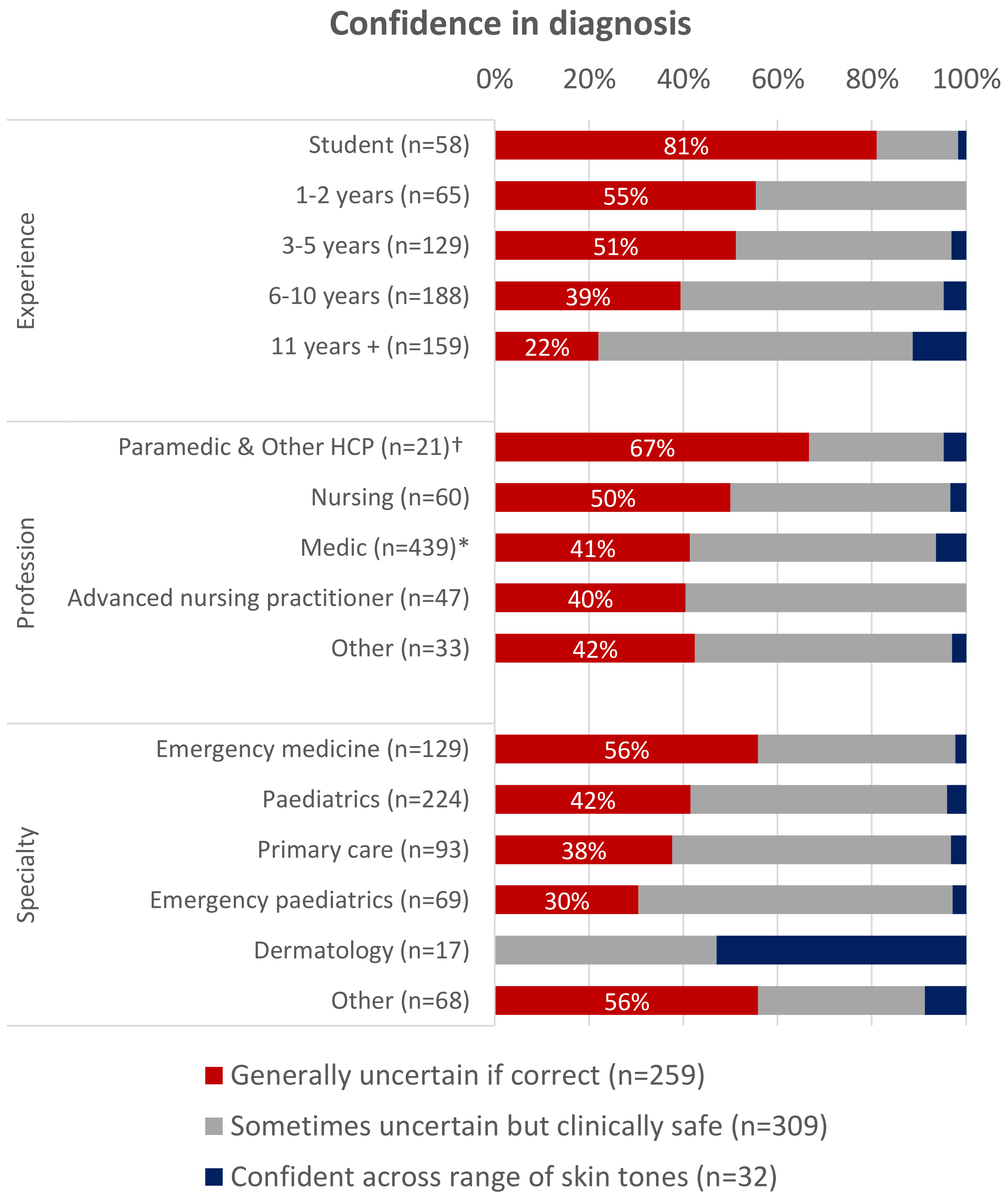

** Including medical doctors/primary care practitioner (all grades) and medical student (n=3), physician associate or assistant (4) and physician assistant-student (1) .*

† Including paramedic (16) and clinical pharmacist (1), pharmacist (1), physiotherapist (1), podiatrist (1) and

Supplementary figure 2. Self-confidence in diagnoses according to experice, profession and specialty.
