# Supplementary material for: Impact of Diversity in Training Resources on Self-Confidence in Diagnosing Skin Conditions Across a Range of Skin Tones: An International Survey
Source: Front Pediatr. 2022 Feb 25;10:837552. doi: 10.3389/fped.2022.837552 (PMC8916608; doi:10.3389/fped.2022.837552)
Supplement: Supplementary file 2 [file Table_2.docx]

| **Table S2 Self-reported confidence by experience, training resources and specialty*** | | | | | | | | | | | | | | | | | | | | | | | | | | | | | | | | | | | | | | | | | | | | |
| --- | --- | --- | --- | --- | --- | --- | --- | --- | --- | --- | --- | --- | --- | --- | --- | --- | --- | --- | --- | --- | --- | --- | --- | --- | --- | --- | --- | --- | --- | --- | --- | --- | --- | --- | --- | --- | --- | --- | --- | --- | --- | --- | --- | --- |
|  | **Experience** | | | | | | | | | | | | | | | | | | | | | | | | | | | | | | | | | | | | | | | | | | | |
|  | **Student (n= 58)** | | | | | | | |  | **1-5 years (n= 194)** | | | | | | | |  | | **6-10 years (n=188 )** | | | | | | | | |  | | | **11 years+ (n= 159)** | | | | | | | | | |  | | |
|  | Clinically safe/ Confident | | | | Generally uncertain | | | | *p-value* | Clinically safe/ Confident | | | | Generally uncertain | | | | *p-value* | | Clinically safe/ Confident | | | | Generally uncertain | | | | | *p-value* | | | Clinically safe/ Confident | | | | | Generally uncertain | | | | | *p-value* | | |
|  | n | | row% | | n | | row% | |  | n | | row% | | n | | row% | |  |  | n | | row% | | n | | | row% | |  |  |  | n | | | row% | | n | | | row% | |  |  |  |
| **Majority Training Resources** |  | |  | |  | |  | |  |  | |  | |  | |  | |  | |  | |  | |  | | |  | |  | | |  | | |  | |  | | |  | |  | | |
| Lighter skin tones | 4 | | 9% | | 41 | | 91% | | *0.001* | 57 | | 40% | | 84 | | 60% | | *0.001* | | 83 | | 58% | | 59 | | | 42% | | *0.281* | | | 84 | | | 75% | | 28 | | | 25% | | *0.160* | | |
| Mixed or darker skin tones | 7 | | 54% | | 6 | | 46% | |  | 35 | | 66% | | 18 | | 34% | |  | | 31 | | 67% | | 15 | | | 33% | |  | | | 40 | | | 85% | | 7 | | | 15% | |  | | |
| **Specialty** |  | |  | |  | |  | |  |  | |  | |  | |  | |  | |  | |  | |  | | |  | |  | | |  | | |  | |  | | |  | |  | | |
| Emergency Medicine | 1 | | 10% | | 9 | | 90% | | *0.669* | 9 | | 30% | | 21 | | 70% | | *0.038* | | 19 | | 40% | | 29 | | | 60% | | *0.001* | | | 28 | | | 68% | | 13 | | | 32% | | *0.082* | | |
| Paediatrics, Primary care, Emergency paediatrics, Dermatology, Other | 10 | | 21% | | 38 | | 79% | |  | 83 | | 51% | | 81 | | 49% | |  | | 95 | | 68% | | 45 | | | 32% | |  | | | 96 | | | 81% | | 22 | | | 19% | |  | | |
| **Data were regrouped based on results from CHAID analysis. Shaded areas indicate smaller sample size in subgroups (n<15).* | | | | | | | | | | | | | | | | | | | | | | | | | |  | |  | | |  | | |  | |  | | |  | |  | | |  |
|  | |  | |  | |  | |  |  | |  | |  | |  | |  | |  | |  | |  | |  | | |  | |  | | |  | | |  | |  | | |  | |  | |
